# The Biomedical Limitations of Magnetic Nanoparticles and a Biocompatible Alternative in the Form of Magnetotactic Bacteria

**DOI:** 10.3390/jfb16070231

**Published:** 2025-06-23

**Authors:** Natalia L. Paul, Rahela Carpa, Rodica Elena Ionescu, Catalin Ovidiu Popa

**Affiliations:** 1 Materials Science and Engineering Department, Faculty of Materials and Environmental Engineering, Technical University of Cluj-Napoca, 400641 Cluj-Napoca, Romania or natalia.paul@stm.utcluj.ro (N.L.P.); catalin.popa@stm.utcluj.ro (C.O.P.); 2 Light, Nanomaterials and Nanotechnology (L2N) Laboratory, CNRS EMR 7076, University of Technology of Troyes, 12 Rue Marie Curie, CS 42060, CEDEX, 10004 Troyes, France; elena_rodica.ionescu@utt.fr; 3 Eut+ Institute for Nanomaterials & Nanotechnologies EUTINN, European University of Technology, European Union; 4 Department of Molecular Biology and Biotechnology, Faculty of Biology and Geology, Babes-Bolyai University, 1, M. Kogalniceanu Street, 400084 Cluj-Napoca, Romania

**Keywords:** nanoparticles, nanotechnology, magnetotactic bacteria, applications, nanomedicine

## Abstract

Nanotechnology has an increasing impact and a great potential in various biological and medical applications. Magnetic nanoparticles (MNPs) stand out for their unique properties, a reason why they have a varied spectrum of applicability in different sectors of activity; in this paper we focus on the medical field. Magnetotactic bacteria (MTB) are a group of Gram-negative prokaryotes that migrate in one direction or another under the influence of an external magnetic field and are a category of microorganisms that constitutively perform the biomineralization of magnetic nanoparticles in the cytoplasm. This review focuses on the general and particular characteristics of magnetotactic bacteria in close correlation with their utility in the medical field, starting with the medical applications of magnetic nanoparticles and arriving at the potential role in nanomedicine of MNPs extracted from MTB.

## 1. Introduction

**Figure 1:**
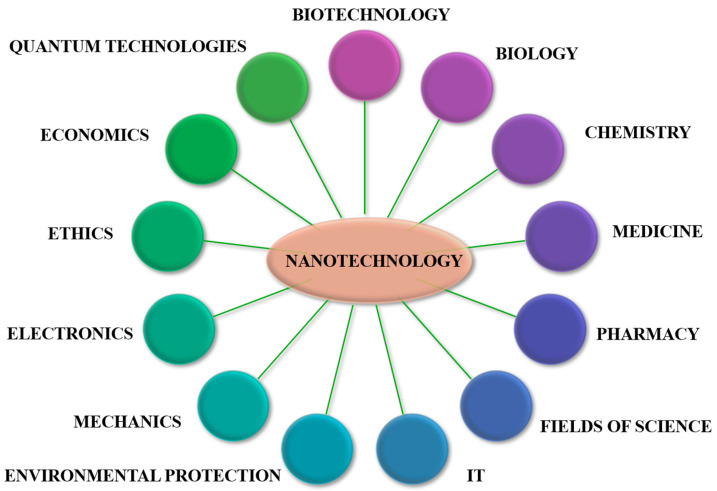
Schematic representation of multidisciplinary interests of nanotechnology.

Nanomedicine aims at the use and administration of nanostructures in an innovative way that allows for progress and new approaches in the development of therapies or diagnostics. Nanoparticles (NPs) and nanostructured materials (NSMs) currently have a significant impact in almost all fields of industry and society, turning them into a point of multidisciplinary interest (Figure 1) [1]. In the medical field, they have earned a leading place through their qualities and the possibility of adjusting their properties according to needs [2]. They have a great potential to be used intensively in medical applications for diagnosis, therapy, and theranostics, such as drug delivery systems, magnetic resonance imaging (MRI) and contrast imaging agents, vaccines, tissue engineering, biomolecule detection, and cancer therapy [3].

Numerous articles have magnetotactic bacteria (MTB) as protagonists. The present work analyzes the general and particular characteristics of magnetotactic bacteria with the aim of revealing those defining features that give them an important place in the development of imaging techniques and therapies in nanomedicine. Hereby we want to reveal the special properties of MTB, the formation of magnetosomes, the molecular mechanisms of biomineralization, and the obtaining and subsequent use of biomineralized magnetic nanoparticles.

## 2. Magnetotactic Bacteria

### 2.1. General Considerations

Magnetotactic bacteria (MTB) were first identified in 1963 by Salvatore Bellini and then rediscovered separately in the late 1970s by Richard Blakemore. The last discovery was made possible by the curiosity directed towards the microbial populations subjected to natural enrichment from the sulfide-rich mud samples and led to the observation of some details that later constituted the source of numerous projects, research works, and new discoveries [4,5].

Despite their seemingly inherent simplicity, bacteria are one of the most abundant types of organisms present in nature and predate other organisms in terms of ancientness. Bacteria differ from eukaryotic organisms by numerous characteristics, which make them some of the simplest found in nature. Compared to eukaryotic organisms, bacteria do not possess the genetic material confined in the nucleus, but they are free in the cytoplasm and possess complex mechanisms of synthesis, reproduction, orientation, and survival. Among these characteristics is their ability to form intra- and extracellular granules of iron hydroxides, phosphates, sulfates, and sulfides [6].

MTB constitute a heterogenous group of prokaryotic organisms; they are Gram-negative microorganisms that are distinct from morphological, phylogenetic, and physiological points of view [6,7,8]. Morphologically, MTB have varied cell shapes (rod, coccoid, ovoid, helical, spiral, or vibrio) and may even show multicellular forms. Regardless of the form, the mobility of MTB is given by the presence of flagella, and the structure of the cell wall is characteristic to Gram-negative bacteria. The movement of the flagella causes movement in the direction imposed by the Earth’s geomagnetic fields as well as by externally applied magnetic forces [7,8,9].

### 2.2. The Presence in Nature and the Behavior of MTB

**Figure 2:**
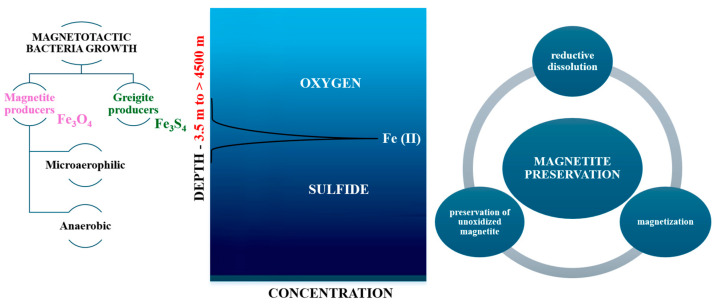
A schematic representation of the chemical gradients and the optimal depth for the growth of various types of MTB.

MTB have a ubiquitous distribution; they are found all over the world, on all continents, in fresh, brackish, marine, and hypersaline waters; they develop in sediments or chemically stratified water columns, located at the oxic–anoxic interface (OAI) (Figure 2) [7,10]. Their presence is not dependent on high concentrations of iron in the habitat, but on the presence of an oxic–anoxic interface that generates gradients of oxygen at the surface and reduced compounds in the sediments or in the water column (especially reduced sulfur compounds) [10]; they have increased density variability in different environments and have distinct depth preferences within vertical gradients, and only some strains have a density with close dependency upon the availability of soluble iron [11].

Magnetotactic bacteria can be easily identified in samples collected from natural habitats, as they have some common, specific attributes: they are Gram-negative, motile, flagellated bacteria, they metabolize short-chain organic acids as carbon sources, they possess magnetosomes, and they have a negative tactic or growth response, consistent with oxygen concentrations. They can contribute to the biogeochemical cycle of several elements (carbon, iron, nitrogen, or sulfur) and have the ability to detect environmental changes, especially changes in oxygen concentration, and thus adapt their direction [9]. Magnetotaxis and chemotaxis, the result of the sensitivity of the bacterial culture to the chemical environment, are imperative aspects in the process of characterizing MTB movement. Magnetotaxis are closely related to chemotaxis, the two ensuring the possibility of MTB to locate and maintain an optimal position of chemical concentration gradients [7,9].

### 2.3. Magnetosomes: Structure, Formation, Biomineralization

**Figure 3:**
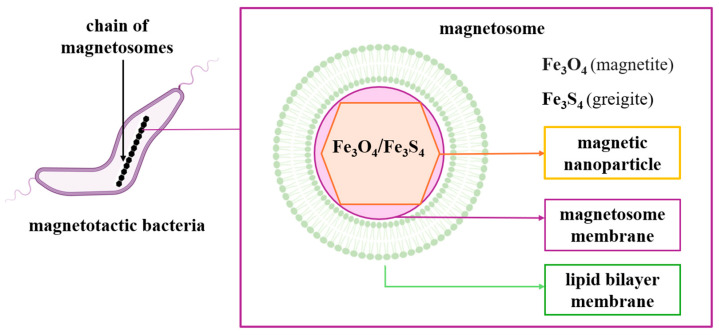
Schematic representation of magnetotactic bacteria and magnetosome chain.

Magnetotactic bacteria convert iron sources into magnetic nanocrystals in the form of magnetite or greigite [4]. MTB produce two types of minerals: iron oxides and iron sulfides. Bacteria that produce iron oxides only biomineralize magnetite (Fe_3_O_4_), and those that produce iron sulfides biomineralize greigite (Fe_3_S_4_) [6]. They have a lipid bilayer membrane that surrounds the nanoparticle core, forming the organelle called the magnetosome. The magnetosome is a distinctive feature of magnetotactic bacteria from other prokaryotic organisms [6] (Figure 3).

Another distinctive feature is given by the arrangement of magnetosomes, along the long axis of the cell, as dispersed aggregates, or localized on one side of the cell [11]. Thus, these are specialized organelles that allow for orientation along the lines of the magnetic field. Magnetosomes contain membrane-encased magnetic crystals arranged in chain-like structures and allow the cell to passively align itself along magnetic fields (magnetotaxis) [5,12]. Morphology, chemical composition, and crystal size are under genetic control.

**Figure 4:**
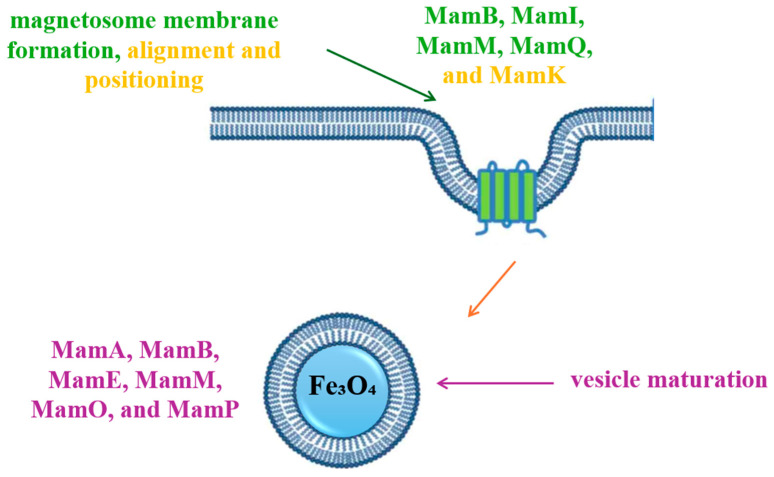
Schematic representation of magnetosome biogenesis. MamB, MamI, MamM, MamQ—key proteins involved magnetosome membrane formation; MamK—key protein involved in alignment and positioning of magnetosome membrane; MamA, MamB, MamE, MamM, MamO, MamP—proteins involved in vesicle maturation.

Magnetosome biogenesis includes four successive stages: the invagination of the cytoplasmic membrane, the sorting of magnetosome proteins on the membrane, the transport of iron in the invaginated membrane and mineralization in the form of magnetic crystals, respectively, the assembly of the magnetosome chain (Figure 4) [13,14]. Following the analysis of the MTB genome, the genes responsible for the formation of magnetosomes were identified, and following the proteomic analysis of the translated gene products, they were grouped into four classes of conserved genes. They were clustered in a hypervariable region called the magnetosomal island (MAI) [15].

Synthesis in magnetosomes resides in the complex processes of the biomineralization of magnetic crystals, composed of magnetite or greigite, which are subsequently assembled into structures with ordered, chain-like shapes. The shape and size of the particles are characteristic elements of each species. Transmission electron microscopy or high-resolution transmission electron microscopy techniques are commonly used to study crystal morphology and structure. The most common forms are those with bullet-shaped, cubic, octahedral, rectangular, or elongated morphologies [12,15,16,17,18,19].

Biomineralization involves the synthesis of minerals through specialized biochemical processes that involve the absorption of ions from the environment, their accumulation and transport in the body, and later mineralization in the necessary structure; this mineralization process ensures physical resistance, protection, and the ability to support the vital functions of the organism [20]. A remarkable biomineralizing element is iron, which, unlike other metals, can form magnetic minerals. MTB attach iron in the form of magnetic nanoparticles that create a magnetic dipole that ensures MTB orientation depending on the magnetic field. This also reduces random movements and ensures the preservation of direction at the oxic–anoxic interface [21]. Iron fixation by MTB has been long studied, especially in the *Alphaproteobacteria* species, *Magnetospirillum magneticum* AMB-1, *Magnetospirillum magnetotacticum* MS-1, and *Magnetospirillum gryphiswaldense* MSR-1 [21].

**Figure 5:**
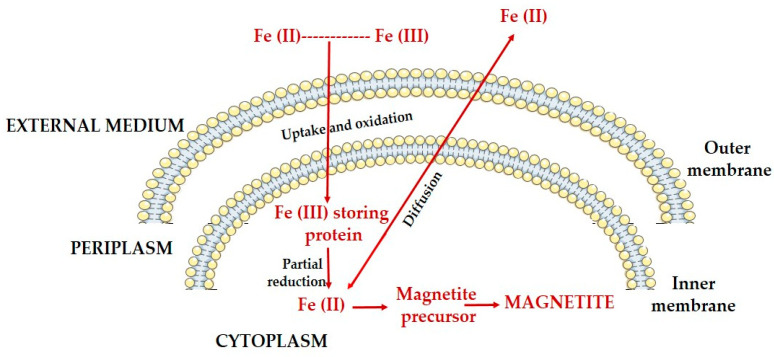
Schematic representation of iron biomineralization in magnetotactic bacteria; uptake of iron from the external environment and conversion into magnetite crystals.

MTB can take up from their environment either Fe[II], whose incorporation is by diffusion, or Fe[III], whose assimilation involves energy-consuming mechanisms. The latter process involves the synthesis of intermediate compounds, which will be eliminated in the external environment, for the solubilization and incorporation of trivalent iron. Given the availability of both forms of iron in the environment, MTB preferentially incorporate divalent iron as a result of direct assimilation, without energy consumption [22,23] (Figure 5). Divalent iron is completely oxidized following the assimilation process, while trivalent iron is stored in cells in cytoplasmic or periplasmic stores (ferritin, bacterioferritin, and DNA-binding proteins) immediately after incorporation [24].

In order to have a comprehensive picture of the mechanisms of magnetosome formation and the optimization of bacterial cultures for biomedical or nanotechnological applications, it is necessary to understand redox transformations, but especially how to quantify the ionic forms absorbed and subsequently converted by MTB.

The scientific literature describes complementary methods that allow for the qualitative and quantitative analysis of iron ions that can be used or extrapolated in the context of their absorption by MTB. Thus, the first such method is UV–VIS spectroscopy with ferrozine for the determination of divalent iron ions. Ferrozine forms a colored complex with divalent iron (intense violet or reddish-purple), thus allowing the differentiated quantification of the latter (wavelength of 562 nm) [25].

Another method that can be used in this regard is electron paramagnetic resonance (EPR), sensitive for the detection of paramagnetic species, especially trivalent iron due to its unpaired electron. In the context of MTB, the EPR technique can allow the identification of the oxidation state of iron, implicitly the indirect conversion of divalent iron to trivalent iron (due to the fact that divalent iron is EPR-silent in most biological configurations) [26].

A feasible option in this context is the use of X-ray absorption spectroscopy, which allows for the determination of the oxidation state of iron in biological samples [27,28], or electrochemical methods due to the possibility of providing complementary information to spectroscopic methods, under dynamic conditions [29,30].

### 2.4. Cultivation of Magnetotactic Bacteria

The cultivation of MTB for the production of magnetic nanoparticles has been a long discussed and studied aspect. Growing MTB under laboratory conditions requires extremely complex and laborious work due to the fact that these bacteria prefer chemically stratified aquatic habitats. Also, another difficult aspect to obtain in the laboratory is maintaining oxygen concentration in optimal parameters, which is critical for the proliferation of MTB. Differences in oxygen concentration, either too low or too high, inhibit bacterial growth. Consequently, only a limited number of bacterial strains have been successfully cultivated under laboratory conditions [31].

Even though the identification of MTB is relatively simple in samples collected from natural environments, they are demanding prokaryotic microorganisms in terms of isolation and cultivation conditions in culture media. All MTB species are microaerophilic, anaerobic, or both [7]. A significant majority are microaerophiles, grow chemolithoautotrophically, and use sulfur compounds, which they reduce, as electron sources. Another part are chemoorganoheterotrophs using organic acids to provide their source of electrons and carbon. Cultivated species have nitrogenase activity and fix atmospheric nitrogen, and many of them denitrify [32].

Depending on the environment from which they were taken, MTB can survive under laboratory conditions for weeks or even years. Optimal storage conditions in the laboratory are at room temperature (approximately 25 °C) and away from strong light to avoid the proliferation of photoautotrophic organisms that can alter or even eliminate magnetotactic bacteria [8]. The identification of MTB from samples, collected from different aquatic environments, is possible due to the magnetic behavior that can be highlighted by the suspended drop technique; this involves, as the name suggests, suspending a drop of sediment or water on a slide and observing it under an optical microscope under the action of a magnet placed in the vicinity of the drop [10,11,32]. Conventional methods of collection and detection of these bacteria are selective for those cells that are highly motile, are in high concentration, and are tolerant for limited periods to atmospheric oxygen concentrations.

Magnetotactic bacteria were enriched by storage in dim light at room temperature, a process lasting up to several months. During the enrichment, morphotypes of magnetotactic bacteria were identified. In the course of studying MTB, it was found that magnetosomes remain, in some cases, stable in sediments in regions where MTB were in high concentrations, even after their death and lysis, leading to a change in the magnetization of the remaining sediments [10,11].

Many studies in this direction have highlighted a wide range of applications of these bacteria [15,20,33,34]. The successful use of MTB requires the determination of their concentration in the culture medium. The concentration is strongly influenced by the short lifetime of active MTB, and the most reliable data is obtained by investigating their magnetic properties, as stated [35]. Researchers found that magnetotactic bacteria are sensitive to chemoattractants only under conditions of direct contact; otherwise, they are insensitive. They respond to these chemical agents, to which they are either attracted or repelled, by random movements given by the rotations of the flagella [33,36,37].

Researchers also explained how upon application of a magnetic field, the magnetosomes align in the direction imposed by this external field [38,39]. But if chemical attractants are added to the environment, depending on their concentration gradient, the bacteria will preferentially migrate in their direction; both bacterial cells and attractants undergo Brownian motion. Thus, in the case of a homogeneous distribution, the researchers concluded that the cells can be forced to deviate from the direction imposed by the magnetic field as a result of the fact that chemotaxis intervene as a resistance factor under conditions of direct contact with the chemoattractant agent [35].

### 2.5. Characterization of Magnetotactic Bacteria

Investigating the properties of magnetotactic bacteria is crucial for their use in biomedical applications. The most common characterization methods refer to the magnetic character of these bacteria using magnetic tweezers (*Magnetosprillum gryphiswaldense*, *Magnetospirillum magnetotacticum*) [40,41], transmission electron microscopy [42], magnetic force microscopy (MFM), or atomic force microscopy (AFM) [43,44,45]. These can provide additional information regarding the structure, morphology, or mechanical and magnetic properties of MTB.

**Figure 6:**
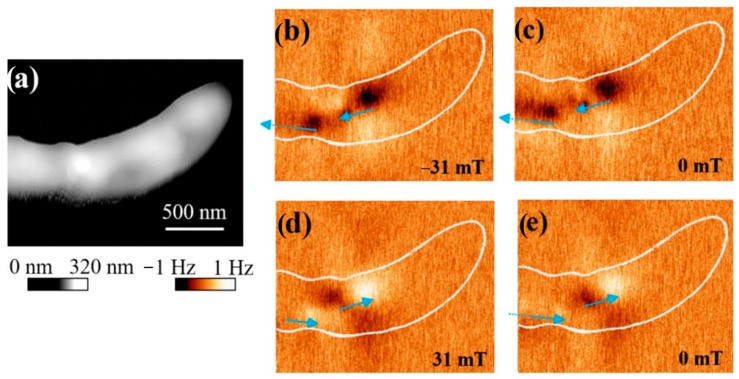
Images of the bacterium *Magnetospirillum gryphiswaldense* MSR-1 obtained by AFM (**a**) and MFM (**b**–**e**); blue lines indicate the direction of magnetization of the magnetosome chain. Reprinted from Ref. [45].

A recent study [45] demonstrated in their study the utility of MFM in the investigation of individual MTB and evaluated the magnetization reversal of magnetosome chains in *Magnetospirillum gryphiswaldense* MSR-1 strains. MSR-1 strains were cultured in standard medium (DMSZ: DMS 6631), at 28 °C, harvested by centrifugation, and washed with PBS [46]. Suggestive images were obtained, allowing us to visualize the stray magnetic field of magnetosome chains by using iron-based magnetic probes in combination with an externally applied magnetic field (Figure 6) [45].

Possible errors or artifacts that may occur in MFM, as stated by by the authors of this study [46], refer to the electrostatic charge present on the MTB surface. In the experiment, the researchers found that the electrostatic potential led to an attraction or repulsion type interaction between the tip and the sample and concluded that the imaging of magnetosome chains inside the MTB is difficult to achieve with standard MFM probes, suggesting a customization of the probes [45].

**Figure 7:**
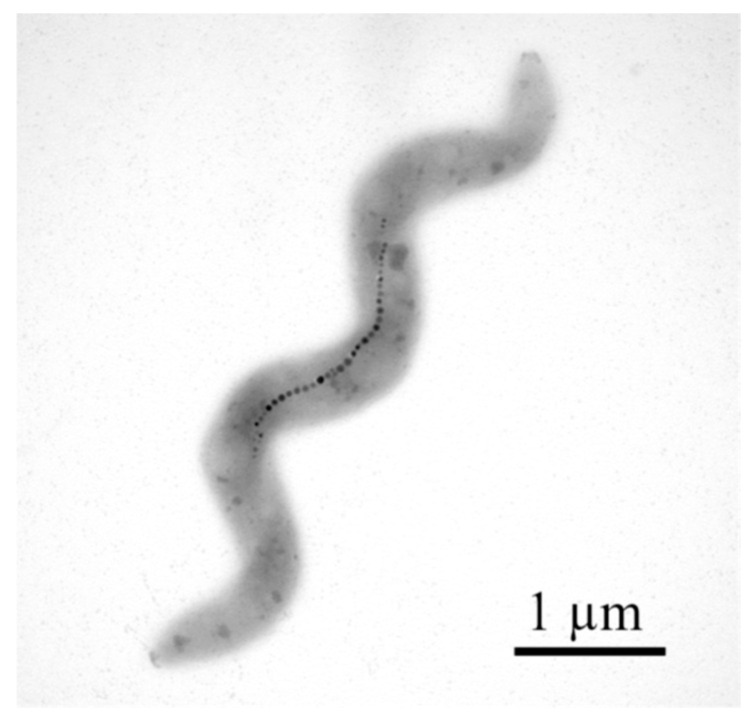
TEM image of magnetotactic bacteria. Reprinted from Ref. [45].

In another study [47], researcherssuccessfully used AFM to obtain high-resolution images of subcellular structures of *M. gryphiswaldense* MSR-1 and *Magnetospirillum magneticum* AMB-1. MSR-1 bacteria were grown in standard culture medium under microaerobic conditions at room temperature [46]. After a few days of growth, they used simple magnets to isolate and concentrate MSR-1 from the culture; the samples were then fixed on a carbon-coated copper grid and characterized by TEM and AFM [45]. TEM allows for obtaining high-resolution images of ultrafine features, while AFM captures the association between proteins and minerals in their natural environment. In this regard, researchers have proposed and succeeded in using AFM to observe the real-time growth of magnetite in the presence of magnetosome-associated proteins (Mms, specifically Mms6) [45] (Figure 7).

**Figure 8:**
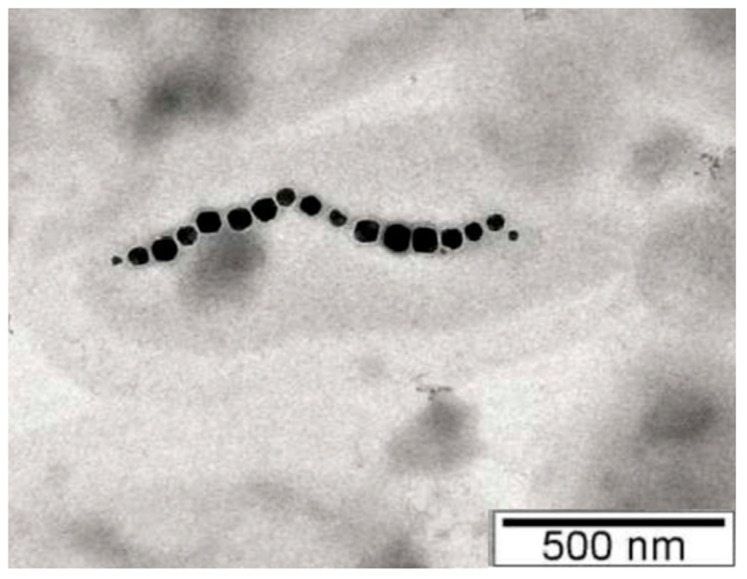
Morphology of *M. magnetotacticum* bacteria with magnetosome chain and the cubo-octahedral shape of magnetite crystals obtained using TEM imaging. Reprinted with permission from Ref. [41]. Copyright 2018 Institute of Electrical and Electronics Engineers.

Another group of researchers [41] used the strain *Magnetospirillum magnetotacticum* (DSMZ 3856) grown on the medium specified by the manufacturer’s website (DSMZ 380 Magnetospirillum Medium). After incubation, they obtained three categories of samples: one category was represented by the bacterial population, another was enriched with bacteria with magnetosomes, and the third was a sample of magnetosomes recovered after cell lysis. In order to analyze the bacteria by AFM, the samples were placed on a mica plate. Their surface was then immobilized with cleaved silica wafers to prevent the molecules from breaking due to the contact with the AFM tip. The TEM images obtained by the authors revealed that only a low percentage of bacteria in the selected population had chains of magnetosomes. AFM imaging, on the other hand, allowed for the highlighting of cellular structures with magnetic behavior. In order to investigate the magnetic behavior more closely, in relation to nature and morphology, the authors subjected the bacteria to cell lysis by sonication [41] (Figure 8).

The observations revealed the presence of magnetic characteristics of magnetotactic bacteria, but after the lysis of the bacteria and recovery of the constituents, they noticed the presence of long and flat structures. The authors concluded that these protein structures actually represent the magnetosome filament, which has an extremely important role in the intracellular positioning of the magnetosome. This leads to the indication that these filaments have magnetic behavior and suggest their possible application in devices with magnetic sensors at the nanometric scale [41].

## 3. Magnetic Nanoparticles: Biofunctionalization and Medical Applications

Magnetic nanoparticles have a high popularity and addressability among nanotechnological processes and applications due to their structural and physicochemical properties, magnetic composition, and, of course, nanoscale dimensions. Functionalized MNPs contain two major components, a magnetic material (iron, cobalt, nickel) and a chemical component with analytical functionality [48,49].

### 3.1. Biofunctionalization of MNPs

The synthesis and functionalization of MNPs have been and continue to be the source of numerous and extensive research. Synthesis methods are essential in shaping decisive aspects regarding morphology, size, and magnetic properties [50]. A critical aspect regarding MNPs is the application of surface functionalization, which directly influences their chemical and physical properties and outlines the potential applications for which they are intended [49,50].

**Figure 9:**
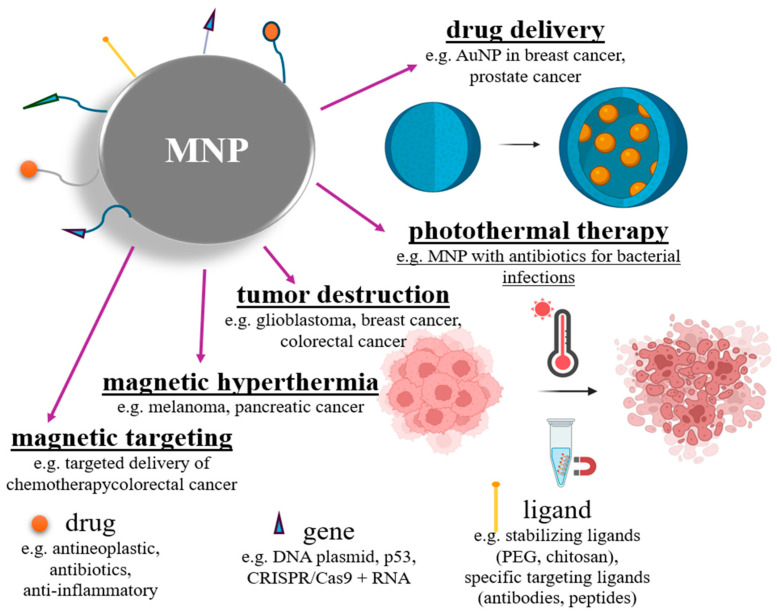
Schematic representation of functionalization of magnetic nanoparticles and potential derived applications.

The main purpose of nanoparticle functionalization is to cover their surface with a molecule that possesses the appropriate functionality required for the designed application. The most commonly used functional groups are amino, biotin, streptavidin, carboxyl, and thiol groups. For many biomedical applications, magnetic nanoparticles (such as magnetite or gold nanoparticles) must be functionalized in order to conjugate with biological entities such as DNA, antibodies, and enzymes [51] (Figure 9).

Some of the most successful methods of interaction of nanoparticles with biomolecules are based on absorption, encapsulation, and bioconjugation [52,53]. The studies carried out in the direction of attaching therapeutic proteins to nanoparticles have obtained promising results due to the low toxicity and biodegradability of proteins, which exhibit distinctive functions in biological materials; they are stable and have a half-life that can be improved by mechanisms that provide the necessary protection to the drug against early enzymatic degradation [54].

In order to determine the biocompatibility of a material, the first step is to analyze the phenomenon of protein absorption on the surface. The mononuclear phagocytic system quickly recognizes the proteins attached to the synthetic materials, and the nanoparticles are phagocytized; macrophages do not recognize the nanoparticles directly, but rather the proteins bound to their surface [53]. The interaction between magnetic nanoparticles and proteins is also the decisive factor that establishes their biological identity. The protein crown that forms around the nanoparticle can impact its behavior, implicitly the biological effect [55].

Proteins are amphipathic molecules with non-specific adhesion to the surface of biomaterials. A thin protein layer minimizes MNPs adhesion and aggregation and consequently avoids their recognition by macrophages. Thus, designing a surface covered with proteins resistant to absorption by the other opsonins to increase the half-life of NP in the blood circulation constitutes a desirable strategy in biomaterials engineering [55]. The protein absorption strategy on the MNPs surface must be studied intensively depending on the specifics of the application, because its success depends on factors such as the type of nanoparticles, the method of administration, and above all on the nature of the proteins to be absorbed. Another fundamental aspect is the knowledge of how these structures interact with other blood proteins, and in this sense, surface plasmon resonance techniques, atomic force microscopy, or isothermal titration calorimetry can provide valuable information [53].

Bioconjugation is another way of attaching proteins to nanoparticles, and its implementation has brought vast improvements in cellular and molecular biology. The selection of the type of bioconjugation is strictly dependent on the physicochemical and biochemical properties of the proteins and the magnetic nanoparticles. Hydrophobicity, site affinity, and MNPs charge influence conjugation, and proteins can interact with the same nanoparticles or with multiple nanoparticles due to capping ligands [56].

Covalent coupling is one of the most commonly used and popular conjugation methods. This method is based on sulfhydryl, carboxyl, amino, and hydroxyl groups of proteins. Proteins can be chemically coupled with magnetic nanoparticles that must be functionalized with the mentioned functional groups (carboxyl, amino, sulfhydryl, hydroxyl) [56].

There are numerous crosslinking agents available that can be chosen according to the specific needs of the application: chemical specificity, degree of cleavage, and spacer arm length.

The length of the spacer arm impacts the contact between the protein and the nanoparticle surface. Zero-length cross-linking agents allow for the establishment of covalent bonds without the insertion of an external spacer but may cause changes in reactivity with proteins in solution and may promote protein denaturation [56].

Biotin-conjugated proteins can be easily attached to the surface of magnetic nanoparticles via the streptavidin–biotin complex, the avidin–streptavidin–biotin bond being the strongest non-covalent interaction. Covalent bioconjugation involves coating MNPs with functional groups, the chemical activation of specific reducing agents, the removal of excess reducing agents, and post-conjugation procedures involving the removal of excess unbound proteins [56].

A disadvantage of bioconjugation is that it can affect the structure and function of the protein, causing its partial denaturation. Protein denaturation during handling can be conformational or chemical. The conformational one can also be produced during the bioconjugation process, and the chemical one is often necessary to obtain coupling with increased efficiency [57].

In situations where proteins do not have the appropriate residue for conjugation, it can be achieved by chemical introduction of sulfhydryl groups. This alters the natural disulfide bonds, causing conformational denaturation and partly chemical denaturation of the protein.

Carboxyl groups play a role in the catalytic activity of enzymes, and their modification further induces conformational changes in the secondary and tertiary structure of proteins. Amino groups are characterized by high reactivity and a lower degree of impact on protein properties [57].

The encapsulation of therapeutic molecules based on proteins, peptides, or enzymes is a strategy to prevent their denaturation. They are susceptible to the action of proteases, and encapsulation is a method of improving the pharmacokinetics of therapeutic substances. The efficiency of encapsulation methods depends on the properties of the administration system, its biocompatibility, its biodegradability, and its ability not to produce immunological reactions in the body [53].

The direct use of MNPs brings with it significant disadvantages, especially related to cytotoxic or mutagenic effects; they cause changes in the structure and function of cells associated with the occurrence of numerous diseases. Undesirable interactions at the MNPs’ surface are also associated with the denaturation of target proteins, which interferes with the safety and efficiency of therapies and biomolecular applications. Magnetic nanoparticles play an important role in various therapies, applications, or diagnostic or treatment strategies. For optimal functioning in the biological environment, their coating is necessary in order to provide a shell that protects the MNPs from the effects of reactive oxygen species, bioactive compounds, and the corrosiveness of the environments they pass through.

### 3.2. Applications of MNPs in the Medical Field

**Figure 10:**
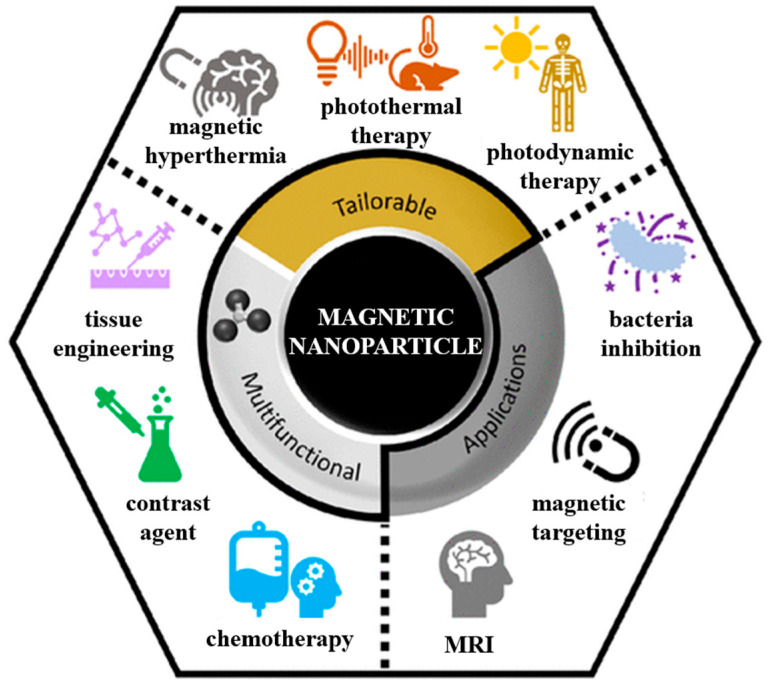
Schematic representation of biomedical applications of magnetic nanoparticles. Reprinted with permission from Ref. [66]. Copyright 2021 American Chemical Society.

Magnetic nanoparticles have a wide range of biotechnological uses in applications such as biomedicine, drug delivery, magnetic resonance imaging, cancer theranostics, biosensors, environmental and agricultural applications, catalysis, and bioseparation [18] (Figure 10). As examples of MNPs used in this sense, we mention iron oxide nanoparticles functionalized with antibodies or peptides for targeted cancer cell imaging and therapy [58,59]; silica-coated magnetic nanoparticles used for biosensing, bioseparation, or enzymatic immobilization [60,61]; chitosan-coated magnetic nanoparticles in drug and gene delivery systems [62]; or nickel ferrite (NiFe_2_O_4_) nanoparticles for controlled drug delivery and tumor hyperthermia [63,64].

Advances in nanotechnology have found utility in therapeutic drug delivery for the treatment of various conditions or disorders. The substance of interest, to be administered, is dissolved and/or attached to stable or biodegradable nanoparticles designed to effectively absorb/adsorb the drug and protect it from enzymatic degradation in order to be delivered to the site of interest with a precise dose [65].

In order to be used in drug delivery, nanoparticles must meet certain criteria. The ideal qualities of MNPs used in this regard are the high drug loading capacity, reduction in the number of matrices used for administration, particle size, and size distribution. All these characteristics will impact on body distribution, toxicity, ability to target the region or site of interest, drug release, and stability of the nanoparticle itself. Drug loading of NP can be achieved by incorporation or absorption/adsorption. Drug loading and entrapment depends on its solubility in the matrix, polymer composition, molecular weight, and polymer–drug interactions [67]. The surface properties of MNPs can be adjusted to achieve the desired solubility, to enhance absorption and biocompatibility. Drug delivery systems can be synthesized in such a way that there is control over composition, shape, size, and morphology. This use of MNPs has numerous advantages; they allow the administration of small molecule drugs, but also of some classes of biomacromolecules, such as proteins, peptides, or DNA plasmids. There are, however, certain shortcomings; they appeal to suboptimal bioavailability, limited site-of-interest targeting for substrate release, and potential cytotoxic effects [68].

Magnetic nanoparticles are used as labels for the targeted detection of analytes in bioassays (streptavidin-coated magnetic nanoparticles, gold-coated magnetic nanoparticles, enzyme-functionalized MNPs) [69,70,71]. Depending on the nature of the biological targets, the test devices are of two types: protein-based and DNA-based. In the case of DNA-based bioassays, the DNA sequence of interest is immobilized on the sensor surface by marking it with MNPs. This functionalization allows the use of MNPs as labels for the evaluation of gene expression of interest [50,72]. Compared to the latter, protein-based tests include a wider range of approaches with a sensitivity directly related to the chosen method, respectively, to the proposed purpose: direct, indirect, sandwich, or competitive [50].

To address the growing concerns about the safety of MRI contrast agents (based on gadolinium and Manganese), their toxicity, potential long-term negative effects, and their accumulation in the body, especially in the brain, a new noninvasive technique called magnetic particle imaging (MPI) has been developed. It involves the direct detection of the electronic superparamagnetization of iron oxide nanoparticles (SPIONs) [50,73]. The MNPs-based technique offers high quality in terms of spatial and temporal resolution and solves the main problem, the issue which triggered this technique; namely, it does not cause side effects [74]. This aspect is supported by studies showing improved biosafety of these particles, compared to currently widely used contrast agents (especially gadolinium) [75,76,77].

Despite the fact that SPIONs have significant potential for use as contrast agents in MRI (due to their superparamagnetic properties), their use in clinical practice is hampered by important limitations. Imaging artifacts (especially at high concentrations, where they can induce edge artifacts and signal distortions), potentially cytotoxic effects (at sizes smaller than 5 nm, they generate reactive oxygen species), difficulties in quantification and non-specific tissue biodistribution (the T2 signal response to increasing SPION concentration is not linear, and signal saturation occurs rapidly, making it difficult to accurately assess the concentration of nanoparticles in tissues), and variability in performance constitute major obstacles to their clinical use [77,78,79,80].

Another successful application of MNPs in the medical field is their use in hyperthermia, an anticancer therapy method in which nanoparticles with high absorption of electromagnetic radiation are administered to the area of interest and excited with a laser beam or a high-frequency magnetic field [81] in order to destroy cancer cells by increasing the temperature of the nanoparticles [82,83]. Also, in the field of anticancer therapies, MNPs have been used for the detection of circulating cancer cells (CTCs). This has been made possible by microfluidic devices for high-sensitivity CTC profiling, in which MNPs are functionalized with target antibodies [74].

Magnetic hyperthermia is a process of controlled heating of magnetic nanoparticles with the aim of generating tumor necrosis. Tumor treatment by hyperthermia involves the induction of thermal stress (between 41 and 46 °C) to destroy tumor cells. Randomized heating causes damage to healthy tissues in the vicinity of the tumor with extremely severe side effects [84,85,86].

Despite their multiple advantages and imperative utility, magnetic nanoparticles currently face multiple shortcomings and disadvantages that limit their use and impose the need to move towards other more biocompatible and sustainable variants in terms of their use for biomedical purposes. In this regard, we recall some of the main shortcomings related to the use of MNPs: instability and agglomeration in vivo, limited targeting of specific tissues, increased risk of toxicity, limitations related to accumulation in the body, reduced efficiency in hyperthermia, and low degree of biocompatibility [87,88,89,90,91].

The list of medical applications of magnetic nanoparticles is long and comprehensive; the short list we presented is directly related to the potential developments using MNPs mineralized in magnetotactic bacteria.

## 4. Medical Applications of Magnetotactic Bacteria

**Figure 11:**
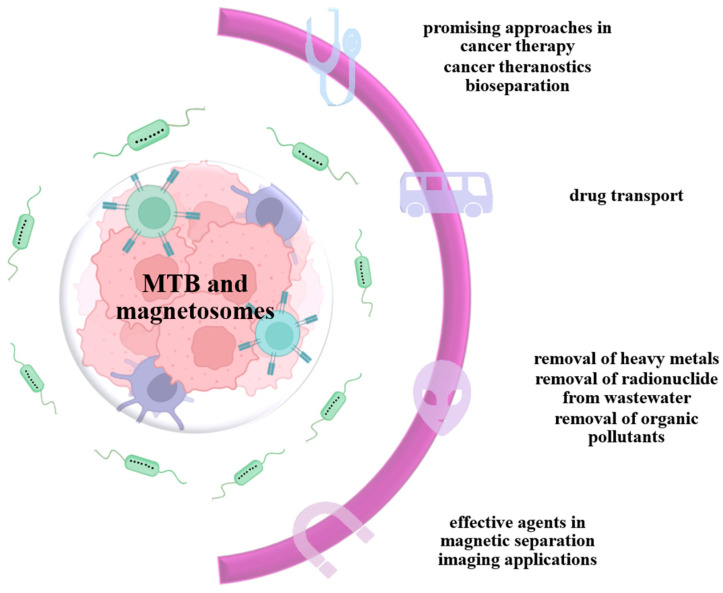
Applications of magnetotactic bacteria MTB and magnetosomes.

The discovery of NPs in magnetotactic bacteria was a revolutionary event and the source of many studies conducted in this direction. Magnetic nanoparticles obtained from magnetotactic bacteria have biotechnological uses in applications such as drug delivery, MRI imaging, cancer theranostics, bioengineering, and bioseparation [18] (Figure 11).

The novel properties, crystallinity, magnetic properties, lack of toxicity, and stability of MTB nanoparticles, make them preferred over NPs obtained by state-of-the-art synthetic routes. MTB-NPs have a very good dispersion in water due to the lipid coating, compared to synthetic NPs [92].

The biofunctionalization of MNPs is of great interest for biomedical applications. The lipid layer of magnetotactic bacteria provides protection to nanoparticles and allows for the immobilization of biomolecules such as proteins, peptides, or DNA through various bioconjugation techniques [93]. Thus, MNPs biomineralized in MTB are presenting significant advantages compared to synthesized MNPs in view of medical applications.

Biotinylation is a bioconjugation method successfully used for MTB-NPs. Biotinylated magnetosomes induce the immobilization of streptavidin and can serve as a bridge for the introduction of another biotinylated species due to the four identical binding sites of streptavidin [93].

Another method of functionalization, unique to MTB-NPs, is genetic manipulation and proteomic techniques that made it possible to sequence the magnetosome membrane (MM) protein. Functionalization in this case involves fusing the MM protein with a reporter gene that functions as a receptor and favors the immobilization of the proteins of interest. These are selected from MM, protein A, GFP, and luciferase genes, as the most commonly used reporter proteins. Fusion proteins can also serve as an indicator in assessing the metabolic activity of magnetotactic bacteria [4,94].

**Figure 12:**
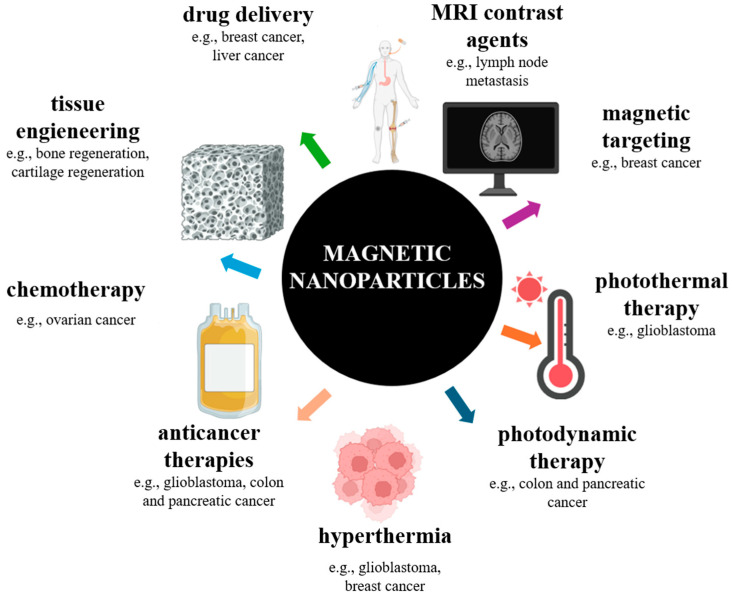
Schematic representation of biomedical and biotechnological applications of functionalized magnetosomes.

An increasing number of studies consider magnetotactic bacteria for their use in the generation of biofunctionalized magnetic nanotubes and nanoparticles. By modifying the membrane surface, magnetosomes can be functionalized either by chemical modification in a dispersed state, or by genetic manipulation of the MM proteins. In this way, macromolecules such as proteins, peptides, or DNA can be functionalized with magnetosomes, thus opening a varied spectrum of possibilities for the use of MTB [95]. These properties give MTB the necessary characteristics to be used in several biomedical applications (Figure 12): drug carriers, hyperthermia treatments [96], contrast enhancers in magnetic resonance imaging [97], and the removal of heavy metals and radionuclides from wastewater by magnetic separation [98].

### 4.1. MTB and Magnetosomes in Drug Delivery and Anticancer Therapy

MTB are considered promising tools in targeted cancer therapy. Their use as carriers of substances with antitumor activity in combination with ligands that recognize specific molecular targets could bring significant benefits to the body by stopping the effects on healthy cells and directing the effects in the microenvironment of cancer cells [99,100]. Due to their properties, bacteria can be considered ideal candidates in anticancer therapies.

One of these considerations appeals to the genome of these bacteria, which allows a high degree of modification and is therefore a promising tool in overcoming the barriers imposed by the treatments currently used. A significant shortcoming of traditional anticancer therapeutics is that they affect both cancer and healthy cells. The promising advantage of MTB is the possibility of targeted delivery of therapeutic agents, but their viability in cancer therapy lies in their behavior towards normal cells. Thus, a key aspect is the monitoring of magnetosome cytotoxicity towards non-tumor cells [100].

A highly desirable aspect of anticancer therapy is the design of efficient delivery systems for therapeutic compounds. The treatments currently used, radiation therapy and chemotherapy, are effective methods, but they also destroy healthy tissues and thus cannot completely destroy cancer cells. Another aspect that prevents the success rate of chemo and radiation therapy is the resistance of cancer cells to multiple drugs.

**Figure 13:**
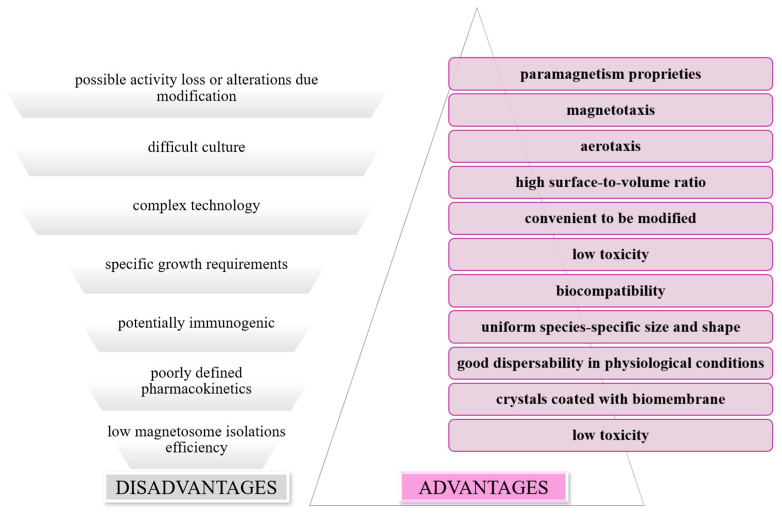
Schematic representation of benefits and limitations of using MTB as drug carriers.

Magnetotactic bacteria are newcomers in medicine, but they are promising approaches in cancer therapy. Magnetosomes can be used as carriers of anticancer therapeutic substances and can be combined with ligands that specifically target cancer cells, thus achieving a focused therapy, avoiding the effects on healthy tissue [101]. Some of the possible benefits but also limitations of the use of these bacteria can be found in Figure 13.

Revathy et al. evaluated the cytotoxicity of magnetosomes both in vitro, in cell lines, and in vivo, in mice. The cell lines used were human erythrocytes and leukocytes, mouse macrophages (cell line J774), onion root tip, and fish (*Oreochromis mossambicus*); the MTB species they used was *Magnetospirillum gryphiswaldense* (MSR-1). The obtained results emphasized the fact that magnetosomes are not toxic, but they pointed out the need for detailed long-term studies of magnetosome toxicity to be able to reveal the possibility of using them in such applications [102].

Another team of researchers highlighted the possibility of targeting MTB to specific pathways in the human body. As a result of the directed mobility of bacteria in the presence of a magnetic field, by changing the magnetic field, it would be possible to control the movement of MTB and direct them in the tumor microenvironment. They could penetrate cancerous tissue, but some cellular connections could be a barrier to their penetration. A viable solution in this regard is the encapsulation of magnetotactic bacteria, a strategy that would improve the safety of transport to the target cells [103].

**Figure 14:**
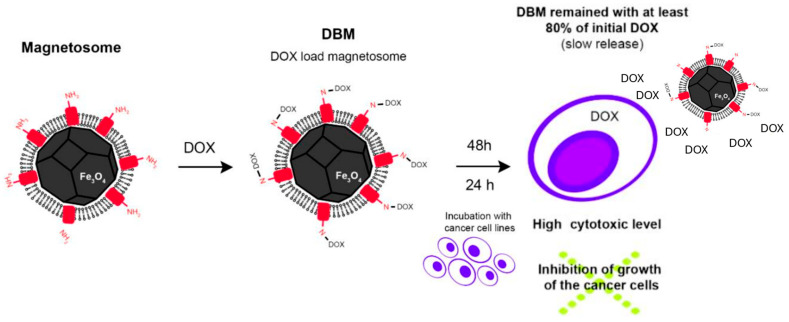
Illustrative scheme of functionalization of magnetosome with doxorubicin (DOX). Reprinted from Ref. [106].

Another team [104] loaded, by covalent attachment, doxorubicin [DOX] onto MTB-NPs to evaluate the potential of these particles to inhibit tumor growth. In their experiment, an efficiency of up to 86% of this administration was observed compared to DOX administered alone. They admitted the possibility of using MTB-NPs as a means of drug transport and the possibility of manipulating them so that therapeutic effects can be manifested at specific sites (Figure 14) [104]. In similar studies, researchers have used daunorubicin and cytarabine with various magnetosome encapsulation protocols. In the experiment conducted by Long et al. (2016), daunorubicin achieved an encapsulation percentage of 36.1%, and the drug loading capacity was 17.9% [105]. Comparatively, in the case of cytarabine, the maximum encapsulation efficiency was obtained at a percentage of 68.4%, and the maximum amount of drug loading was 38.9% [106].

In the evaluation of the obtained results, comparable concentrations of free drugs and drugs attached to the magnetosome were found, and the antitumor activity of the complex was similar to that of free cytarabine. However, the long-term release profile of the drug from the cytarabine–magnetosome complex was remarkable, so the researchers found a gradual release, over 40 days of incubation, of up to 90% of the drug loaded in the complex. The stability observed by them is suggestive and emphasizes the need for a lower number of doses of conjugated cytarabine in order to administer the treatment [106].

Gene therapy is another field of application of these bacteria. This involves the regulation of some essential transcription factors in tumor progression, respectively, the silencing of some genes; MTB can serve as vectors due to their biological and physicochemical properties, thus providing promising solutions and directions in cancer therapy [107].

The potential of using MTB in drug delivery has been demonstrated by the studies conducted in this direction. The development of a product to be successfully applied in medical applications, especially in theranostics, must include a wide range of attributes related to physicochemical characterization, toxicity evaluation, biocompatibility, pharmacokinetic evaluation, and large-scale reproducibility. Nanoparticles must be examined both in vivo and in vitro throughout their development, and their approval for medical use by regulatory agencies involves a laborious task that requires a lot of dedication, effort, and investment [8].

### 4.2. Magnetosomes in Hyperthermia

An alternative to the usual methods (radio and chemotherapy) used in the treatment of cancers is hyperthermia. This method is often attractive due to the fact that it is less restrictive, compared to radiotherapy and chemotherapy, and has fewer toxic effects; it can be used in combination with the usual methods, thus increasing the chances of the treatment being effective [108]. Hyperthermia acts by increasing the temperature inside the tumor and destroying the cancer cells, obtaining a reduction in the volume of the tumor or even its complete elimination [109,110,111].

The nanoparticles used in these therapies are artificially synthesized and are often limited in terms of efficacy due to the reduced ability to be specifically placed and to exclusively target the tumor tissue. Thus, superparamagnetic nanoparticles are accompanied by side effects when administered in the body [108]. In this case, magnetosomes are also distinguished as effective systems, in hyperthermia, by the possibility of selective localization at the tissue level; they are a highly promising alternative to superparamagnetic nanoparticles and, in addition, by using an alternative magnetic field, allow for adjusting the amount of drug released from the functionalized magnetosomes [111,112].

In this sense, Alsaiari et al. used strains of *Magnetospirillum gryphiswaldense* to evaluate the control of substrate loading and release processes using gold nanoparticle-conjugated ssDNA. They found an increased efficiency of internalization into the bacterial cell through endocytosis by macrophages, and the conjugated molecules [ssDNA-AuNPs] were released by magnetic hyperthermia [113]. By exposing the magnetosomes to an alternating magnetic field, heating can be achieved, either by reversal of the magnetic moment or by the rotational motion of the magnetosomes under the magnetic field. Magnetosomes have properties clearly superior to synthetic magnetic nanoparticles in terms of behavior at high temperatures; they have a uniform magnetic moment at any temperature [96,109].

### 4.3. MTB and Magnetosomes as Magnetic Resonance Contrast Agents

Another use of MTB is as molecular probes in nuclear magnetic resonance. A comparative study [114] revealed that bacterial magnetosomes and synthetically obtained magnetic nanoparticles can be used as negative contrast agents. These have strong effects in T2-weighting, with the specification that magnetosomes showed superior signal attenuation to synthetic counterparts [114].

Nanoparticles from magnetotactic bacteria have distinct superior properties compared to many other types of nanomaterials, but their use in imaging applications such as MRI is constrained by production limitations. The researchers also found that the transfected cells have the potential to use endogenous iron resources in the magnetite synthesis process and the MamA protein can be used as a reporter gene that has the ability to transform any cell into a small magnetite factory [115].

MTB-NPs can also serve as imaging probes, having the advantage of higher sensitivity compared to other conventionally used probes. Researchers [115] used these bacteria to label macrophages for the purpose of detecting inflammation, and the characterization was performed using MRI. Macrophages were labeled ex vivo and injected into mice suffering from peritonitis. MRI analysis revealed an accumulation of macrophages in areas of high inflammation, particularly in the colonic region [115].

The authors of another research study [114] tested magnetosomes in cell culture and animal models to demonstrate their potential to be used as contrast agents [116]. The obtained results revealed their utility in MRI imaging of brain, pancreatic, tumor cell, and mammalian cell models. Studies concluded that magnetosomes have proven to be useful tools in the detection and treatment of tumors by hyperthermia as a result of their high affinity for target cells [117,118].

Moreover, we consider that, in certain applications, MTB could be used in molecular spin probes. This use could provide information about local magnetic field fluctuations, about the interactions between spin probes and magnetosomes, and also about the dynamics of the magnetosome membrane and the topology or arrangement of crystals in the magnetosome chain.

### 4.4. MTB and Magnetosomes in Cell Separation

Early research on magnetosomes demonstrated that they could be used in cell separation. Magnetosomes have been successfully used in the separation of leukocytes, based on their phagocytosis profile; after their exposure to a magnetic field, a concentration of up to 95% of leukocytes was found, demonstrating an efficient separation of cells fused with magnetosomes compared to un-complexed ones [119].

Other researchers have performed magnetic separation of cells using magnetosomes and specific antibodies; magnetosomes were bound to immunoglobulin G to separate peripheral blood cells (*CD*14^+^ monocytes, *CD*19^+^ and *CD*20^+^ B lymphocytes). They found that inserting a protein with an immunoglobulin-binding domain on the magnetosome surface simplifies the process of their functionalization [120].

### 4.5. MTB and Magnetosomes in the Control of Pathogens

The specific properties of magnetosomes from magnetotactic bacteria have also found use in the capture of pathogens such as *Salmonella* species, *Vibrio parahaemolyticus*, or specific enterotoxins of *Staphylococcus aureus* [121,122,123].

A group of researchers [122] succeeded in isolating some *Salmonella* species from food; they were able to detect and separate *Salmonella dublin* from a test suspension with a capture efficiency of 87%. These results allowed them to extend their research to artificially contaminated food samples: milk, eggs, and pork. The recorded findings revealed a detection and capture of the pathogen when it was present in concentrations greater than 60 UFC/mL [122].

Similarly, a different group of researchers [123] made a recombinant complex using the protein A gene fused to the *MamC* gene of the magnetosome from *Magnetospirillum gryphiswaldense* MSR-1 strain cells. This complex was linked to a specific antibody in order to detect and capture *Vibrio parahaemolyticus*, a gastrointestinal pathogen whose transmission is carried out through food, from insufficiently heat-treated food. This complex allowed for the capture of 1.74 × 10^7^ pathogen cells [123].

*Staphylococcus aureus* enterotoxin was identified in contaminated milk using a biosensor composed of the magnetosome complex conjugated with anti-enterotoxin antibodies specific to this pathogen. The biosensor had a detection threshold of 0.017 ng/mL and a high capacity to measure pathogen concentration [121].

### 4.6. MTB and Magnetosomes in DNA/Antigen Detection Assays and Enzyme Immobilization

Magnetosomes have found utility as components in protein, antigen, or DNA detection assays. In this regard, a group of researchers [12] used antibody-functionalized magnetosomes to immobilize hepatitis B antigen (HbsAg) from human serum. Antigen detection was possible by using an automated immuno-PCR technology, the sensitivity of which was up to 100 times higher compared to the ELISA (enzyme-linked immunosorbent assay) detection technique [12].

Enzyme immobilization can be achieved using magnetic nanoparticles and magnetosomes, the latter demonstrating a simple recovery process by magnetic separation. To test and demonstrate this, in biofuel production, the researchers used a multienzyme complex attached to the surface of magnetosomes via a peptide bridge. The obtained results indicated good performance of the magnetic nanoparticles regarding the reuse of the enzymes of interest (cellulase) as a result of the fact that more than 70% of the cellulose degradation activity of the enzyme complex was preserved after five reuse cycles [124].

## 5. Conclusions

MNPs play an essential role in modern medicine, with applications as diverse as medical imaging, gene therapy, targeted drug delivery, and magnetic hyperthermia. Existing applications, both in research and those already implemented clinically, demonstrate a formidable potential in improving the efficiency of treatments, as well as diagnosis. MTB-NPs’ applications are much more promising compared to those of synthetic MNPs, mainly due to their improved characteristics in terms of biocompatibility, functionalization, and sustainability. MTB are often functionalized with proteins, peptides, or antibodies that specifically target cells or tissues and reduce the incidence of adverse effects.

The advantages of using MTB-NPs are varied, but among the most important we mention biocompatibility, reduced risk of toxicity and immunological rejection, increased stability, and extended functionality in numerous therapies, treatments, or devices.

In terms of future applications, MTB-NPs could become an essential tool in personalized medicine, with the potential to revolutionize treatments through precise drug delivery, tissue regeneration therapies, or even gene therapies. However, continued research and improved manufacturing technologies are crucial in minimizing the risks and maximizing the benefits of these nanomaterials.

This review focused on general and specific features of magnetic nanoparticles from magnetotactic bacteria in order to examine them for use in biomedical applications.

The essence of this work lies in the correlation between the limitations of the use of magnetic nanoparticles (which are indispensable in many fields of activity, especially in biomedicine) and directing the reader, respectively, the researchers, towards a variant, in the form of magnetic nanoparticles mineralized by magnetotactic bacteria, which offers the promise of more sustained biocompatibility and which can fill the gaps in the form of alternatives to the methods currently used. Therefore, we placed the emphasis and tried to draw a line of direct correlation between magnetic nanoparticles and the more sustainable and biocompatible variant offered by magnetotactic bacteria.

## Data Availability

No new data were created or analyzed in this study. Data sharing is not applicable to this article.
